# The Gibbs Fundamental Relation as a Tool for Relativity

**DOI:** 10.3390/e27010074

**Published:** 2025-01-15

**Authors:** Friedrich Herrmann, Michael Pohlig

**Affiliations:** Faculty of Physics, Karlsruhe Institute of Technology, 76128 Karlsruhe, Germany; pohlig@kit.edu

**Keywords:** relativistic thermodynamics, entropy, chemical potential, Gibbs fundamental relation

## Abstract

When relativistic physics is lectured on, interest is focused on the behavior of mechanical and electromagnetic quantities during a reference frame change. However, not only mechanical and electromagnetic quantities transform during a reference frame change; thermodynamic and chemical quantities do too. We will study the transformations of temperature and chemical potential, show how to obtain the corresponding transformation equations with little effort, and exploit the fact that the energy conjugate extensive quantities, namely entropy and amount of substance, are Lorentz-invariant.

## 1. Introduction

When introducing relativity and when discussing reference frame changes, usually the primary concern is the behavior of physical quantities from mechanics or electromagnetism, which are particularly significant for particle physics and cosmology. However, not only mechanical and electromagnetic variables transform during a reference frame change; thermodynamic and chemical quantities do too. In the following discussion, we will explore the transformations of temperature and chemical potential.

We start from the Gibbs fundamental relation and realize that the transformation behavior is the same for the intensive quantities temperature, chemical potential, and electric potential. The reason for this is that the respective energy conjugate extensive quantities, i.e., entropy, amount of substance, and electric charge, are Lorentz-invariant. We show that the three transformation equations can be obtained with little effort. We do not take the detour via the transformation of the kinematic variables, which often causes confusion for students.

## 2. The Gibbs Fundamental Relation

Let us briefly recall the Gibbs fundamental relation.

(1) dE=TdS−pdV+μdn

(E = energy; T = absolute temperature; p = pressure; V = volume; μ = chemical potential; n = amount of substance).

It is usually introduced in the context of thermodynamics. The quantities behind the differential sign are extensive quantities; those in front are the energy conjugated intensive variables. Keep in mind that this applies to homogeneous systems; there is only one temperature value, one value of the chemical potential, and one of pressure. Furthermore, it only applies in this form if we are dealing with a single substance. If our system was composed of subsystems with different temperatures, pressures, or chemical potentials, the equation would contain additional terms.

What does the equation actually tell us?

- The energy content can be changed in three different ways, namely by changing its entropy, its volume, or its amount of substance. Each term characterizes a so-called exchange form of the energy.
- It can be read as the defining equation of the intensive quantities in front of the differential sign. In fact, it is commonly used to define the scales of the thermodynamic temperature and of the chemical potential.

If only processes are considered in which n is constant, and if we abbreviate TdS=dQ and −pdV=dW, the equation is referred to as the first law, where Q is the exchanged heat and W is the performed work.

In our context, it is important to note that the Gibbs fundamental equation in the form of Equation (1) is only the special case of what is of interest in thermodynamics. By adding further terms, it can describe other types of energy exchange [1,2].

For our purposes, let us add two more terms:

(2) dE=TdS+μdn+UdQ+v·dp

Electric charge Q and momentum p are the extensive variables, and the electric potential difference U and velocity v are the energy conjugate intensive variables. We no longer need the term −pdV because it is contained in the term v·dp as a special case.

For readers who are not familiar with the expanded form of the equation, let us give two examples that illustrate the effect of the last two terms.

- If the charge of a capacitor between whose plates the voltage U prevails is increased by dQ, the stored energy increases by UdQ.
- If the momentum of a body moving with velocity v is increased by dp, the energy stored in the body increases by v·dp.

## 3. The Transformation of Entropy and Temperature When Changing the Reference System

We consider a process where the entropy and none of the other extensive quantities on the right-hand side of Equation (1) are supposed to change in the system at rest (denoted by subscript 0). Equation (2) reduces to

(3) $$dE_{0}=T_{0}dS_{0}.$$

We now look at the same process in a moving reference frame. In this case, a new term appears in the Gibbs fundamental relation. The transferred energy corresponding to the term TdS is equivalent to transferred energy and, according to $E=mc^{2}$, to transferred mass. This means that in the moving reference frame, momentum is also transferred to the considered system. The corresponding Gibbs fundamental equation must now include the term v·dp:

dE=TdS+v·dp,

where dp is the momentum supplied to the system and v is the velocity of the moving reference frame relative to the reference frame at rest. Since dp and v are parallel to each other, we can write the equation with the magnitudes v and dp of the velocity and the momentum

(4) dE=TdS+vdp.

The transferred momentum can be written as

$$dp=d\left(mv\right)=mdv+vdm.$$

Since for our process

dv=0,

we obtain

dp=vdm.

Using

$$dm=\frac{dE}{c^{2}}$$

we obtain

$$dp=\frac{vdE}{c^{2}}$$

and finally

$$vdp=\frac{v^{2}dE}{c^{2}}.$$

Substitution into Equation (4) results in

$$dE=TdS+\frac{v^{2}}{c^{2}}dE$$

and thus

$$dE\left(1-\frac{v^{2}}{c^{2}}\right)=TdS.$$

We abbreviate

$$\beta =\frac{v}{c}$$

and obtain

$$dE\left(1-\beta^{2}\right)=TdS.$$

We now take advantage of the fact that entropy is Lorentz-invariant (see Appendix A), i.e.,

$$dS=dS_{0}.$$

In addition, we know how energy transforms:

(5) $$dE=\frac{dE_{0}}{\sqrt{1-\beta^{2}}}.$$

Thus, Equation (4) becomes

$$dE_{0}\sqrt{1-\beta^{2}}=TdS_{0}.$$

With Equation (3), we finally obtain

(6) $$T=T_{0}\sqrt{1-\beta^{2}}.$$

The temperature in the moving reference frame, seen from the rest frame, is reduced by the factor

$$\sqrt{1-\beta^{2}}.$$

Equation (6) was already derived shortly after the publication of the special theory of relativity [3] (pp. 1–34) [4,5], but it was later questioned. In some papers, another relationship was derived [6] (pp. 70–104), namely

$$T=\frac{T_{0}}{\sqrt{1-\beta^{2}}}.$$

This result is obtained if the term vdp in Equation (4) is not taken into account.

There are other authors [7] (p. 938) who believe that the transformation law of the temperature is a subject of choice. Our thermodynamic derivation shows that such a choice does not exist.

## 4. Taking over the Result to Other Exchange Forms of the Energy

The only characteristic ingredient concerning the term TdS that we have used in the previous calculation was the fact that the entropy is Lorentz-invariant.

This observation has an interesting consequence: all intensive quantities whose energy conjugate extensive quantities are Lorentz-invariant transform in the same way. Among the terms appearing on the right-hand side of Equation (2), these are, in addition to entropy, electric charge and amount of substance.

Therefore, we obtain two more transformation equations

(7) $$\mu =\mu_{0}\sqrt{1-\beta^{2}}$$

and

(8) $$U=U_{0}\sqrt{1-\beta^{2}}.$$

We would like to emphasize that we have obtained Equations (6)–(8) without the detour over kinematics. Also, Equation (5), that we have used, can be derived directly from the energy–mass equivalence [8].

## 5. New Questions and Answers

Since one usually encounters relativistic transformations only in the context of mechanics and electrodynamics, one may wonder where Equations (6)–(8) play a role at all.

One reason to deal with them is to see that physics is coherent across the various sub-areas. The extension of the Gibbs fundamental relation is already a step in this direction.

It may be that the equations do not answer any questions one might have. However, they do suggest new questions and help us to find answers to these questions.

Let us take another look at the three relations (6)–(8) one by one in this respect.

A consequence of Equation (6) has already been discussed in [9]. The fact that the temperature is dependent on the reference frame seems to lead to a paradox, which is resolved in this article.

A paradox can also be formulated with regard to Equation (7).

Bob is on the terrace of the station restaurant, and Alice is on a passing train. It is a hot day. Bob has a glass of iced water in front of him, with the ice finely crushed, resulting in a mixture at 0 °C. Alice has a similar drink. Bob is puzzled that the ice in Alice’s glass is floating in liquid water, because he had learned that according to the theory of relativity, the temperature in a moving reference frame is lower than in a stationary one. Therefore, he believes Alice’s drink should be below 0 °C and frozen. But Alice is equally surprised, expecting Bob’s drink to be frozen. Both assume that the melting temperature is an intrinsic property that cannot change with a reference frame change. End of the story.

The error that leads to the paradox can be recognized if one remembers that the condition for phase equilibrium between solid and liquid water is that the chemical potentials of the two phases must be the same. However, if they are the same in the reference frame in which the substances are at rest, they must also be the same in a reference frame in motion, because both chemical potentials—that of the solid and that of the liquid phase—transform in the same way.

Finally, let us look at the effect of the electric term of Equation (2). We apply it to a charged capacitor, which we first describe in a reference frame in which it is at rest, and then in one in which it is moving with velocity v perpendicular to the plates. Since the charge is a relativistic invariant and using

$$Q=C\cdot U=\frac{\varepsilon_{0}A}{d}\cdot U$$

we can conclude that the plate spacing d transforms in the same way as the electrical potential difference:

$$d=d_{0}\sqrt{1-\beta^{2}}.$$

(C = capacity; $\varepsilon_{0}$ = vacuum permittivity; A = area of the plates; d = plate spacing).

We know the relationship as Lorentz contraction. We have obtained it without taking the usual route via kinematics.

## 6. Conclusions

We have obtained the relativistic transformation equations of absolute temperature, chemical potential, and electric potential with little effort using the Gibbs fundamental relation.

The fact that the three energy conjugate extensive quantities are Lorentz-invariant has the consequence that the corresponding intensive quantities transform in the same way. All three intensive quantities have lower values by a factor $\sqrt{1-\beta^{2}}$ in the moving reference frame than in the system at rest.

Instead of presenting relativity in teaching as a kinematic theory, we recommend placing the emphasis on dynamics and also on thermodynamics. This not only makes relativity easier to understand but also shows that the theory is more comprehensive and not merely limited to kinematic problems.

## Data Availability

The original contributions presented in this study are included in the article. Further inquiries can be directed to the corresponding author.

## Appendix A

We use the fact that the three quantities, *S*, n, and Q, are Lorentz-invariant. Here is a brief reminder of how this can be justified.

- Electric charge

Its value is an integer multiple of the elementary charge e. The elementary charge is a universal constant. The number of elementary charges, i.e., an integer, cannot change continuously with a continuous change in the velocity of the reference frame. It can therefore only remain constant.

- Amount of substance

Its value is an integer multiple of the amount of substance of a single particle (which we could call the elementary amount of substance). The same argument applies as for the electric charge.

- Entropy

Its value S=k·lnΩ is also obtained by pure counting, namely the number Ω of accessible microstates of the system. Therefore, it must also be Lorentz-invariant.
